# Tamarillo Polyphenols Encapsulated-Cubosome: Formation, Characterization, Stability during Digestion and Application in Yoghurt

**DOI:** 10.3390/antiox11030520

**Published:** 2022-03-08

**Authors:** Tung Thanh Diep, Michelle Ji Yeon Yoo, Elaine Rush

**Affiliations:** 1 School of Science, Faculty of Health and Environment Sciences, Auckland University of Technology, Private Bag 92006, Auckland 1142, New Zealand; tung.diep@aut.ac.nz; 2 Riddet Institute, Centre of Research Excellence, Massey University, Private Bag 11222, Palmerston North 4442, New Zealand; elaine.rush@aut.ac.nz; 3 School of Sport and Recreation, Faculty of Health and Environment Sciences, Auckland University of Technology, Private Bag 92006, Auckland 1142, New Zealand

**Keywords:** tamarillo extract, yoghurts, cubosome, polyphenols, encapsulation, in vitro digestion

## Abstract

Tamarillo extract is a good source of phenolic and anthocyanin compounds which are well-known for beneficial antioxidant activity, but their bioactivity maybe lost during digestion. In this study, promising prospects of tamarillo polyphenols encapsulated in cubosome nanoparticles prepared via a top-down method were explored. The prepared nanocarriers were examined for their morphology, entrapment efficiency, particle size and stability during in vitro digestion as well as potential fortification of yoghurt. Tamarillo polyphenol-loaded cubosomes showed cubic shape with a mean particle size of 322.4 ± 7.27 nm and the entrapment efficiency for most polyphenols was over 50%. The encapsulated polyphenols showed high stability during the gastric phase of in vitro digestion and were almost completely, but slowly released in the intestinal phase. Addition of encapsulated tamarillo polyphenols to yoghurt (5, 10 and 15 wt% through pre- and post-fermentation) improved the physicochemical and potential nutritional properties (polyphenols concentration, TPC) as well as antioxidant activity. The encapsulation of tamarillo polyphenols protected against pH changes and enzymatic digestion and facilitated a targeted delivery and slow release of the encapsulated compounds to the intestine. Overall, the cubosomal delivery system demonstrated the potential for encapsulation of polyphenols from tamarillo for value-added food product development with yoghurt as the vehicle.

## 1. Introduction

Inverse bicontinuous liquid crystalline nanoparticles, termed cubosomes, have advantageous properties that may be suitable for the delivery of bioactive compounds to the small intestine. Amphiphilic lipids such as the monoglyceride monoolein can self-assemble in water to produce dispersions of cubosomes. The basic structure of a cubosome is a honeycomb-like structure with two non-intersecting internal aqueous channels separated by lipid bilayers. The internal hydrophilic (aqueous) areas are separated by lipid bilayers that are twisted into a tightly packed three-dimensional honeycomb structure that has a high internal surface area to volume. Within this structure, encapsulation of diverse hydrophilic, hydrophobic and amphiphilic compounds of small to large molecular weights, such as proteins, peptides, amino acids and nucleic acids, is possible [1]. Within cubosomes, hydrophobic molecules can be located within the lipid bilayers, hydrophilic components in the aqueous channels or around the polar head of the lipid, and amphiphilic molecules can be located at the lipid–water interface. This structure generally maintains the efficacy—stability of actives (vitamins and proteins) without adverse effects on the recipient [2]. Both polar and non-polar compounds can be solubilized in the water channel and the bilayers, respectively; therefore, both can be loaded into these particles. According to Meikle et al. [3], cubosomes are relatively non-toxic, stable over a broad range of biologically relevant environmental conditions, and can be formulated using a wide array of lipids and stabilizers. Their size, surface charge, and bilayer structure can be tuned through adjustments in their composition. Moreover, previous studies have demonstrated the ability of cubosomes to deliver drugs to both the eukaryotic and prokaryotic cells [4,5,6,7,8] found in the human digestive system. Cubosome encapsulation has been used to deliver quercetin in vitro [9] and curcumin to the skin [10] and demonstrated improved solubility and availability with an entrapment of 84% and 82%, respectively. Improved anti-inflammatory/antioxidant effects and controlled diffusion of encapsulated curcumin through the skin were observed [10]. Cubosome encapsulated has also enhanced the stability and antibacterial activity of curcumin [11]. Another study had reported successful encapsulation of both piperine and curcumin in the interior of the cubosome particles [12].

Tamarillo (*Solanum betaceum* Cav.), a common fruit of New Zealand, is a good source of polyphenols compounds including anthocyanins, hydroxy benzoic acids, hydroxycinnamic acids, flavonols, flavanols and flavonol glycosides. The main polyphenols in the dried pulp of Laird’s Large tamarillo cultivar were identified in our previous study [13] as delphinidin rutinoside (255 mg/100 g), pelargonidin rutinoside (201 mg/100 g DW), chlorogenic acid (66 mg/100 g), kaempferol rutinoside (50 mg/100 g) and cyanidin rutinoside (26 mg/100 g). These polyphenols are strong antioxidants possessing many potential health benefits such as preventing lipid oxidation and are associated with reduced risk for certain cancers, cardiovascular diseases and type 2 diabetes mellitus [14]. Tamarillo fruit therefore has the potential to be an ingredient in functional food products [15]. Evaluation of the stability of polyphenol compounds is important as these compounds are often degraded by oxidation during digestion, resulting in reduced biological activity [16]. For example, anthocyanins, present in high amounts in the nutrient-dense tamarillo, are oxidized into quinones, reducing the biological power of these molecules during digestion [16]. To overcome this obstacle, new generation–functional foods often use encapsulation technologies to protect polyphenols from degradation as well as maintain their bioavailability [16].

This study aimed to investigate the morphology, entrapment efficiency, and particle size of cubosome-encapsulated tamarillo extract (CUBTAM) and test the stability and antioxidant activity and release of the CUBTAM before and after in vitro digestion. In vitro digestion of yoghurt fortified with CUBTAM was similarly investigated for the stability and release of polyphenol compounds.

## 2. Materials and Methods

### 2.1. Materials

The yoghurt ingredients included standard milk (Anchor™ blue top, Fonterra) from a local supermarket (Auckland, New Zealand) and starter culture containing *Lactobacillus delbrueckii* subsp. *bulgaricus* and *Streptococcus thermophilus* (YoFlex^®^ Express 1.1 powder) from CHR Hansen (Hoersholm, Denmark).

Dimodan^®^ MO 90/D (monoolein) was kindly provided by Danisco (Auckland, New Zealand). Pluronic F127 (PEO_99_–PPO_67_–PEO_99_) was purchased from Sigma-Aldrich (Auckland, New Zealand). The analytical grade standards of phenolics and anthocyanins were obtained from Sigma-Aldrich (Auckland, New Zealand) or Extrasynthese (Genay Cedex, France). Purite Fusion Milli-Q water purifying machine (Purite Limited, Thame, Oxon, UK) was used to produce Milli-Q water. All chemicals and reagents used were AnalaR grade or purer.

### 2.2. Tamarillo Extract (EXT) Preparation and Identtification of Polyphenol Components

Fresh fruits of Laird’s Large (red) tamarillo cultivar were collected from growers in the Northland region of New Zealand with commercial maturity of 21–24 weeks from anthesis. The fruits were cleaned and peeled; then, the pulp was packed in polyethylene bags, sealed and frozen at −18 °C and defrosted (15 min) immediately before use. The extraction process was based on the method of Piovesana and Noreña [17] with some modifications. Aqueous citric acid (2% *w*/*v*) was used for extraction in the ratio of 1:5 (tamarillo: aqueous citric acid, *w*/*w*) and homogenised in a blender, and agitated (1000 rpm) at 55 °C in a water bath (1000 rpm) (Heidoplph, LABOROTA) for one hour after the addition of (20 μL/100 mL) pectin lyase (Novozym 33095), to improve the extraction of the bioactive compounds. Parafilm and tightly sealed lids of container and centrifuge tube were used to prevent entry of air and oxidation of polyphenols. The mixture was centrifuged at 10,000 RPM for 10 min and the supernatant (tamarillo extract) was separated and stored at −20 °C until being injected into LC-MS for identification of polyphenol component as reported in our previous study [13].

### 2.3. Preparation of Cubosome and Cubosome Containing Tamarillo Extract

The lyotropic liquid cubic phase was prepared and dispersed into cubosomes (CUB) as previously described in previous studies [18,19,20]. Briefly, molten monoolein (50 °C) was mixed with MilliQ water at a mass ratio of 60:40 to form the cubic phase. The cubic phase gel was equilibrated at room temperature for at least 48 h. Then, 25 mg of cubic phase gel was added to 5 mL of Pluronic F127 solution (2% (*w*/*v*)), and sonicated using a probe sonicator (BEM-150A, Bueno Biotech Ltd., Nanjing, China) (50% amplitude, pulsing 5 s on, 5 s off, for 7 min total run time) to obtain cubosomes (CUB). In parallel, the water was replaced by the tamarillo extract with a mass ratio of 60:40 (monoolein: tamarillo extract) to produce tamarillo polyphenol loaded-cubosomes (CUBTAM).

### 2.4. Polarized Light Microscopy (PLM) and Scanning Electron Microscopy (SEM)

Microstructure and morphology of CUB and CUBTAM particles were observed using a polarized light microscope (DM750, Leica, Wetzlar, Germany) equipped with a digital camera (ICC50HD, Leica, Wetzlar, Germany). Dried CUB and CUBTAM were coated by using Platinum (Pt) using a sputtering technique with a Sputter Coater (Hitachi E-1045, Hitachi, Tokyo, Japan) for 60 s at room temperature. The particle morphology was then observed by a scanning electron microscope (Hitachi SU-70 Schottky, Hitachi, Tokyo, Japan) at 25 mA and 10 kV [21].

### 2.5. Dynamic Light Scattering (DLS)

Each sample of the CUB and CUBTAM diluted 20× in MilliQ was measured in triplicate using disposable plastic cuvettes at 25 °C. Particle size and its distribution were determined by dynamic light scattering (DLS) using a Zetasizer Nano ZS (Malvern Instruments Ltd., Worcestershire, UK). The mean *z*-average diameter and polydispersity indices (PDI) were obtained from cumulative analysis using the Malvern software version 7.13 (Malvern Panalytical Ltd., Worcestershire, UK) [18].

### 2.6. Determination of the Entrapment Efficiency (EE)

The sample preparation procedure was applied according to Zhou et al. [22] with minor modifications. Briefly, 20 μL of CUB or CUBTAM were transferred into a 1 mL Eppendorf microcentrifuge tube and made up to volume with methanol. Then, the sample solutions were centrifuged at 5000 RPM for 5 min, and the supernatant was separated and transferred to an amber 1.8 mL glass vial, then injected into the LC-MS system.

The phenolic and anthocyanin compounds were analysed by using LC-MS according to our previous study [13] without further modification. According to Patil, Pawara, Gudewar and Tekade [23], the entrapment efficiency (EE%) is calculated as follows:

$$\mathrm{EE}\%=\frac{A-B}{A}\times 100$$

where A is the polyphenol concentration added into cubosome and B is the polyphenol concentration present in the supernatant

### 2.7. Yoghurt Fermentation and Fortification with Tamarillo Polyphenol Loaded-Cubosome

CUBTAM suspensions from Section 2.3 were snap frozen by liquid nitrogen, and then lyophilized for 36 h (Alpha 1–2 LD plus Freeze Dryer, Martin Christ, New Zealand). The tamarillo bioactive loaded-cubosome (CUBTAM) powder was stored at −20 °C until use.

Kitchen yoghurt makers (Davis & Waddell, Stevens, New Zealand) were purchased to produce the yoghurt. For the control yoghurt, starter culture and milk in the ratio of 0.1:100 (*w*/*w*), were placed in the yoghurt maker. Incubation was implemented at 45 °C for 8 h until the pH of below 5.0 was obtained. The yoghurt was stored at 4 °C overnight and then homogenized at 4000 RPM using laboratory mixer (Silverson, Waterside, UK) for 2 min [24]. The yoghurt was stored at 4 °C until further analyses within 24 h.

The CUBTAM powder with concentration of 5, 10 and 15% were fortified into the yoghurt either before (PRE) or after (POS) the fermentation process. For PRE, CUBTAM powder was added to the mixture of milk and starter culture at the start of yoghurt making process, prior to fermentation. For POS, CUBTAM was added to yoghurt in the final homogenization step.

### 2.8. Determination of Physicochemical Properties of Fortified Yoghurts

The pH was measured with a digital pH meter to one decimal place. Syneresis index of yoghurt was identified based on the method of Wang et al. [24]. Some modifications were made from Kristo, Miao and Corredig [25] for rheological measurements, using a rheometer (RST-SST, Brookfield Ametek, Middleboro, MA, USA). The viscosity profile (viscosity, consistency coefficient, flow behavior index) was determined with a concentric cylinder with the diameters of cup and bob of 28.92 and 26.66 mm, respectively. Elastic modulus was determined with a vane spindle (SSVANE-) at a speed of 0.5 rpm for 5 min.

TA-XT plus texture analyser (Texture Technologies Corp., New York, NY, USA) was used to perform textural analysis with a backward-extrusion test based on the method of Wang et al. [24] with some modifications. The parameters of test included cylinder probe diameter of 50 mm, test speed of 1.0 mm/s, penetration distance of 25 mm and surface trigger force of 10 g.

### 2.9. In Vitro Digestion

The EXT, CUB, CUBTAM and yoghurt samples fortified with CUBTAM were subjected to in vitro digestion to identify the bioavailability of bioactive contents using method of Zhang et al. [26] without further modification. The sample (2 mL) was collected before digestion and after oral (5 min), gastric (120 min) and intestinal phases (180 min). The samples were snap frozen using liquid nitrogen to stop enzyme activity and centrifuged at 10,000 RPM at 4 °C for 10 min. The supernatants were collected and stored at −20 °C before being injected into the LC-MS for phenolic and anthocyanins identification as well as total phenolic content (TPC) and antioxidant activity analysis [13].

### 2.10. Total Phenolic Content (TPC) and Antioxidant Activity

The TPC of extracts and digests at each stage of digestion was determined using a Folin–Ciocalteu assay as described in our previous study [13]. Two different methods were used to determine the antioxidant activity, namely cupric ion reducing antioxidant capacity (CUPRAC) and ferric-reducing antioxidant power (FRAP) assays, which were mentioned in our previous study [13]. Results of TPC and antioxidant activity are presented as mg gallic acid equivalent per 100 g of tamarillo or yoghurt (mg GAE/100 g) and μmol Trolox equivalent antioxidant capacity per 100 g of tamarillo or yoghurt (μmol TEAC/100 g), respectively.

### 2.11. Statistical Analysis

Measurements of all the analytes were undertaken in triplicate, and the results are presented as mean ± standard deviation (SD). For comparison among different samples, one-way analysis of variance (ANOVA) was applied using SPSS 25.0 (IBM Corp., Armonk, NY, USA). Fisher’s (LSD) multiple comparison tests were used to determine the magnitude of differences between means. A *p*-value of <0.05 was considered statistically significant.

## 3. Results and Discussion

### 3.1. Characterization of Cubosomal Suspensions Containing Tamarillo Extract (CUBTAM)

Adapting the temperature–composition phase diagram of a monoolein/water system [27], pure monoolein cubosomes (CUB) and tamarillo polyphenols loaded-cubosomes (CUBTAM) were prepared in a top–down approach. This method allows the formation of reproducible, stable cubosomes without adding solvents. Therefore, it is unnecessary to reinvestigate phase behaviour, and there are no solvent concerns for cellular toxicity [2]. The concentration of the surfactant pluronic F127 was chosen to be 2 wt%, which yields stable cubosome dispersions [28]. The CUB dispersion appeared homogenously milky white and CUBTAM appeared semi-opaque pink (picture not shown).

**Figure 1:**
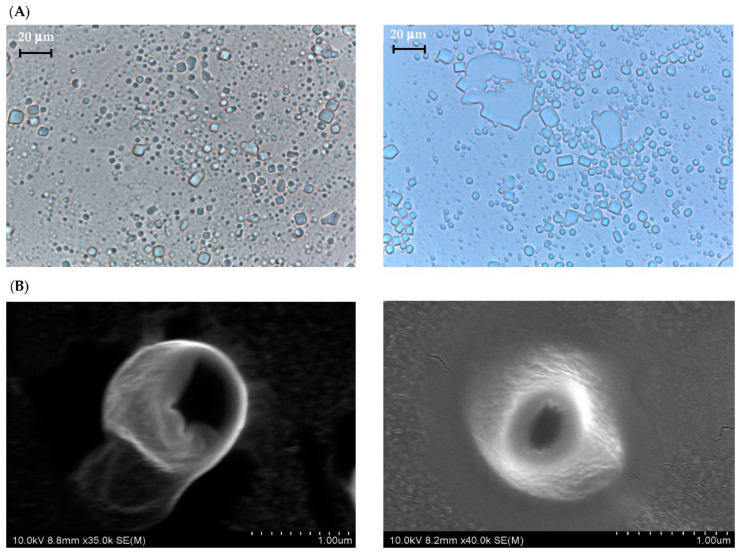
(**A**) PLM and (**B**) SEM micrographs of CUB (**left**) and CUBTAM (**right**).

Figure 1 shows the PLM and SEM photos of CUB and CUBTAM while their particle size distribution (PSD) is summarized in Figure S1 and Table S1. The addition of tamarillo extract did not significantly affect the morphology of cubosome particles. The initial cubic periodicity can be clearly visualized for both samples using PLM and SEM. Because tamarillo extracts are mainly water-soluble compounds (phenolics and anthocyanins), they should be dispersed in the water channel of the cubosome and should minimally affect the structure of the nanoparticles [19].

For particle size distribution, CUBTAM had a unimodal curve and its polydispersity index (PDI) was quite small (below 0.3), as shown in Figure S1. The mean hydrodynamic diameter of liquid crystal particles increased significantly (from 270 to 327 nm) with the addition of the tamarillo extract. In general, this parameter depends on several factors such as the concentration of amphiphile (lipid and polymer), the presence of charged lipids, the ionic strength and the interactions between groups [23]. For CUBTAM, the addition of hydrophilic groups contributed to increasing electrostatic interaction as well as the coalescence between colloidal particles resulting in a bigger average particle size. However, most of the cubic particles in CUBTAM were still limited to a sub-micron range (100–1000 nm). According to Danaei et al. [29] the small particle size and the narrow size distribution (small PDI) create a large surface area that benefits cellular uptake.

**Figure 2:**
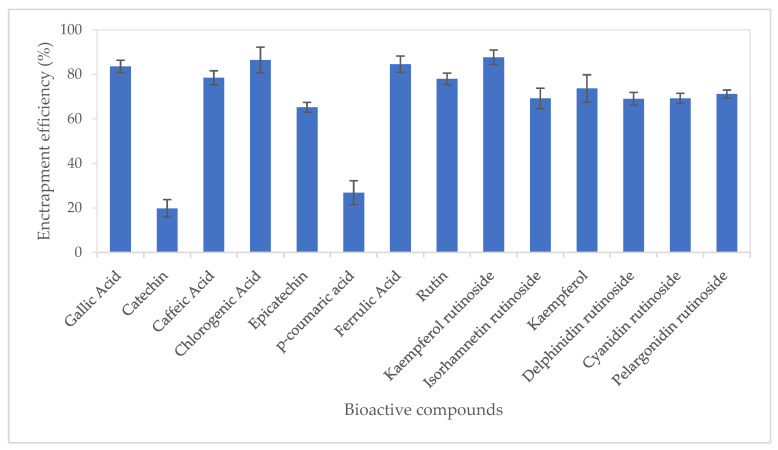
Entrapment efficiency of polyphenols from tamarillo extract using lyotropic liquid crystalline nanoparticles. Data are presented as mean and error bar (standard deviation) (*n* = 3).

Entrapment efficiency (EE%) of bioactive compounds from CUBTAM ranged from 19.8 (catechin) to 87.7% kaempferol rutinoside (Figure 2). Twelve of the fourteen tamarillo bioactive compounds had an EE of more than 50%. In addition, it is noteworthy that we show high EE% (>69%) for the major polyphenols in tamarillo (chlorogenic acid, kaempferol rutinoside, delphinidin rutinoside, cyanidin rutinoside and pelargonidin rutinoside). The high EE in the CUBTAM could be attributed to the fact that polyphenols in tamarillo extract are water-soluble compounds which embed in the water channels. The EE difference between polyphenols encapsulated by cubosome might also depend on the number of -OH groups in molecular structure. For example, the hydroxycinnamic acids chlorogenic acid, caffeic acid and ferulic acid (with >2 -OH groups) showed higher EE than p-coumaric acid (which has only 1 -OH group). More -OH groups will more easily attach in the aqueous channel of cubosome particles. Furthermore, different polyphenol classes showed different EE. For instance, the hydroxycinnamic acids (chlorogenic acid, caffeic acid and ferulic acid), hydroxybenzoic acid (gallic acid), flavonol glycosides (rutin, kaempferol rutinoside, isorhamnetin rutinoside) and anthocyanins (delphinidin rutinoside, cyanidin rutinosid, pelargonidin rutinoside) showed higher EE than flavanols (catechin, epicatechin). According to Patil et al. [23], the EE is dependent on particle size rather than the amount of poloxamer (pluronic F127) used to stabilise the cubosome. The larger the nanoparticles, the higher entrapment efficiency for the polyphenols. This is because surface area to volume ratio of large particles is less than that of smaller particles and exposure to water of active compounds also decreased. Thus, the active compound loss due to diffusion also decreased in larger particles.

### 3.2. TPC and Antioxidant Activities of Encapsulated and Non-Encapsulated Extracts during In Vitro Digestion

**Figure 3:**
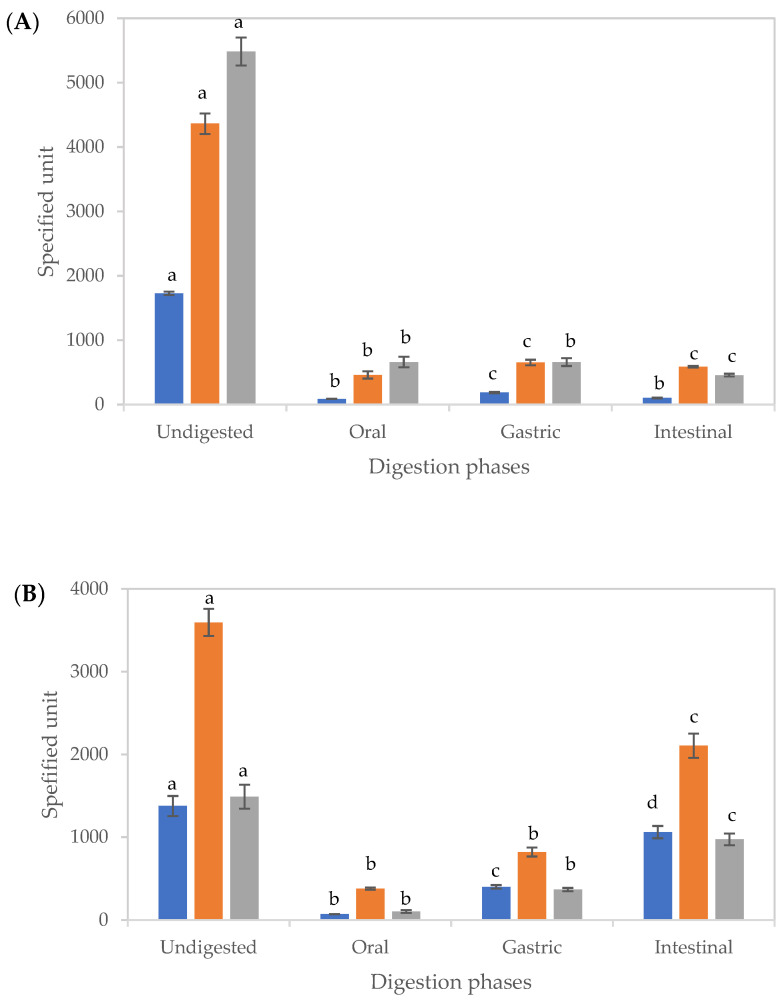
Changes in the total phenolic content and antioxidant activities of tamarillo extract (**A**) and tamarillo polyphenol loaded-cubosomes (**B**) before and after in vitro digestion. The units of TPC (■), CUPRAC (■) and FRAP (■) were mg GAE/100 g tamarillo, μmol TEAC/100 g tamarillo and μmol TEAC/100 g tamarillo, respectively. Data are presented as mean and error bar (standard deviation) (*n* = 3). Different alphabets indicate statistical difference (*p* < 0.05) for each assay.

The impact of digestion on TPC and antioxidant activities of EXT and CUBTAM is shown in Figure 3. The TPC and antioxidant activities decreased significantly after digestion for both non-encapsulated and encapsulated in comparison to the undigested samples.

For both EXT and CUBTAM, there were significant differences (*p* < 0.05) between the amounts of TPC in the supernatant after each stage of digestion. Gastric digests recorded the highest TPC for EXT, while, in the oral and intestinal phases, no significant differences were observed (Figure 3A). Low values of TPC in the supernatant of the oral digest (after 2 min of digestion) are related to the short time for diffusion and low solubility of polyphenols. The loss of polyphenols during digestion could be explained by physicochemical transformations, such as oxidation or the presence of yoghurt molecules (fats and proteins) in the digestion mixture. Furthermore, the decrease of bioactive content could arise from precipitation of several compounds with proteins or enzymes in the digest [30].

However, for the CUBTAM, the release of polyphenols increased during the digestion and was greater in the intestinal phase than the gastric phase (Figure 3B), which was not seen for the non-encapsulated extract, demonstrating a protective effect of the cubosome encapsulation technique against digestive enzymes and pH changes during gastric digestion. Results obtained from LC-MS (Table 1) further support the protective effect of the cubosomes on polyphenols. Similar findings have been reported on release properties of encapsulated blueberry extract [31] and carob pulp extract [32] during simulated gastrointestinal digestion. Both studies showed that TPC in the supernatant increased throughout gastric to intestinal digestion. The materials used for lipid bilayer and stabilisation of cubosomes determine susceptibility of polyphenols to digestive enzymes as well as pH at each stage [33]. The reduction in TPC of tamarillo extract during in vitro digestion might be associated to sensitivity of phenolic compounds to higher pH (>6), since, at that pH, monomers obtained by hydrolysis from larger molecules might be less stable [34]. The increase in the TPC of CUBTAM could be related to the release of complexed bioactive compounds as a result of the digestive process [35].

The antioxidant activity of tamarillo fruit phenolic extracts is mainly linked to their anthocyanins, chlorogenic acid and kaempferol rutinoside compounds. However, due to the chemical transformations from different mechanisms, the antioxidant properties of these compounds might change during digestion. Thus, the influence of digestion on the antioxidant capacity of tamarillo pulp extracts in non-encapsulation and encapsulation form was assessed by using CUPRAC and FRAP assays (Figure 3).

All activities tested significantly (*p* < 0.05) decreased after digestion in comparison to the raw material, which coincide with the decrease in bioactive compounds, mainly polyphenols, after digestion (Table 1). There were substantial and significant differences in CUPRAC values between the non-encapsulated and encapsulated extracts (*p* < 0.05) throughout the process of in vitro digestion. Tamarillo extract had the highest CUPRAC value in the gastric phase, whereas, for CUBTAM, the highest supernatant activity was noted in the intestinal medium. EXT and CUBTAM presented significant differences (*p* < 0.05) in FRAP values during the digestion process. The highest FRAP value was observed in the oral phase for non-encapsulated extract, whereas, for encapsulated samples, this activity increased with the progress of digestion with the highest FRAP activity of the supernatant was recorded at the end of the intestinal phase. In other studies, an increase in the FRAP with digestion was most pronounced at the intestinal phase for both encapsulated blueberry extract [31] and carob pulp extract [32].

The difference in FRAP and CUPRAC activities, in the oral and intestinal phases, respectively, may not be due to the content of phenolics and anthocyanins, but rather to the diversity and characteristics of the polyphenols present. However, the highest activities (FRAP and CUPRAC) after the gastric phase might be due to the higher release of phenolics and anthocyanins content and the quenching and reducing properties of the acidic medium of the sample. The effect of the pH could also be different among various polyphenols. At neutral pH, some polyphenols have exhibited pro-oxidant activities, whereas, at lower pH, others have demonstrated antioxidant activities [32]. Furthermore, the difference might be related to in vitro digestion conditions used and/or change of polyphenol availability related to the release of matrix associated compounds [36]. In fact, free polyphenols have shown higher antioxidant activity than iron-phenol chelates. Together with the enzymatic action, the pH influence within the gastrointestinal digestion and the presence of compounds that were not analysed in this study (e.g., peptides or complex polyphenols) enhance antioxidant activity [35]. The increase in antioxidant power of the supernatant between the acidic gastric phase and alkaline intestinal phase environments, as seen in this study, can be partially explained by the deprotonation of the hydroxyl groups on the aromatic rings of the polyphenols [36].

Under the intestinal conditions, the decrease in antioxidant activity (CUPRAC and FRAP) for the non-encapsulated extract would be related to the lower TPC alongside transformation of some polyphenols into conformations related to the neutral pH (Figure 3A). Meanwhile, the highest antioxidant activities for CUBTAM supernatant, in the intestinal phase (Figure 3B), could be explained by their release from the microcapsules as they are degraded in the neutral pH. The weak activities recorded in oral and gastric phases of digestion might be due to a small amount of polyphenol release from the microcapsule surface and/or via the penetration of salivary and gastric fluids into the microcapsules through their surface pores.

### 3.3. Release of Tamarillo Polypehnols from Cubosomes during Digestion

In order to evaluate the stability of individual polyphenol compounds during digestion, a total of fourteen compounds were evaluated by LC-MS (Table 1). The CUB was also analysed as a control. The results showed that 11 phenolic compounds and three anthocyanins were released from the microcapsules after the digestion process, demonstrating that these phytochemical compounds were well encapsulated by the cubosomes.

The phenolics presented different behaviours during the simulated digestion (Table 1). Analysis of phenolics released from EXT during digestion showed a significant instability for the major phenolic acids (gallic acid, chlorogenic acid and *p*-coumaric acid), other phenolics (epicatechin, rutin and kaempferol rutinoside) as well as all anthocyanins after oral and gastric phases. For gallic acid, the concentrations in oral phase remained stable and only a significant (*p* < 0.05) increase was observed in the gastric phase (46.49%) when compared to the initial undigested EXT. Then, the concentration of this acid dropped down to 20.4%. Tagliazucchi, Verzelloni, Bertolini and Conte [37] reported the degradation (43%) of pure gallic acid after gastrointestinal digestion, while the total degradation for gallic acid from grape extract and carob pulp extract had been explored by Jara-Palacios et al. [38] and Ydjedd et al. [32], respectively. Meanwhile, caffeic acid showed insignificant changes during the digestion (24.13% at the oral phase, 31.42% after the gastric phase and 21.94% at the end of the intestinal phase). According to Wojtunik-Kulesza et al. [39], the remaining percentage of caffeic acid decreased to 75% and 78% after oral and gastric phases, respectively. Some studies have reported that the gastric phase has increased the bioaccessibility of some phenolic acids, while, during the intestinal phase, their levels could be decreased. This behaviour has been closely related to the stability and structural changes that each type of polyphenolic acid undergoes [37]. Due to its low molecular weight, gallic acid has been better absorbed in humans compared to other phenolic acids, which makes it highly bioaccessible [36]. For chlorogenic acid, the highest concentration was detected in the gastric phase; then, the concentration of this compound reduced by 63% in the intestinal phase (Table 1). According to Tagliazucchi, Helal, Verzelloni and Conte [40], the degradation of chlorogenic acid during gastro-pancreatic digestion might be due to the oxidation and polymerization to form quinone in an alkaline environment. Significant reductions of free phenolic acids (gallic, chlorogenic, caffeic, *p*-coumaric acids) during in vitro digestion have been reported in previous studies [41,42,43]. These decreases in phenolic acids could be related to changes in pH and the presence of bile salts in the intestinal phase, which may lead to the formation of precipitates [42], which may explain the reductions observed at the end the intestinal phase of this study.

The concentrations of kaempferol rutinoside in EXT remained stable after the oral and gastric phases but decreased significantly (*p* < 0.05) at the end of the intestinal phase (Table 1). A similar trend for kaempferol rutinoside during in vitro digestion of the Cactus Cladodes plant had been observed [44]. Hydrolysis of glycoside flavonoids starts in the mouth by means of *β*-glycosidase action, but the degree of hydrolysis depends on the types of sugars present in the flavonoid compounds. For example, polyphenol compounds with more hydrophobic properties often interact more strongly with proteins [39]. Degradation of polyphenols with high molecular weights (such as kaempferol rutinoside) may be related to their strong affinities with human salivary proline- and histidine-rich proteins to form non-covalent and covalent associations [39].

All of the anthocyanins, especially delphinidin rutinoside and pelargonidin rutinoside, showed the same releasing behavior during in vitro digestion (Table 1). For these main anthocyanins in tamarillo extract, a significantly (*p* < 0.05) higher proportion of anthocyanins (43 to 76%) was released after the intestinal phase when compared to the undigested samples. The instability of anthocyanins at neutral or slightly basic pH has been observed for polyphenols from grape and chokeberry [37,45]. The instability can be explained by the formation of a colourless chalcone pseudo-base, resulting in the destruction of the anthocyanin chromophore [46]. The current results support these previous findings, suggesting that anthocyanins are stable in the acidic conditions of the gastric phase but are degraded in the alkaline/neutral conditions of the intestinal phase. The reduction of anthocyanins may also be related to the fact that, in aqueous solution in response to changes in pH, anthocyanins undergo structural rearrangements, change colour, may form complexes with proteins in food and digestate and be degraded to phenolic acids [42].

The quantity of individual bioactive compounds from the CUBTAM at the end of each digestive phase varied by compound (Table 1). Catechin, epicatechin, isorhamnetin rutinoside and all anthocyanins (delphinidin rutinoside, cyanidin rutinoside and pelargonidin rutinoside) were released after the gastric phase in acidic medium; gallic acid, caffeic acid, chlorogenic acid, p-coumaric acid, ferulic acid, rutin, kaempferol and kaempferol rutinoside were released into the neutral medium after oral and intestinal phases. It is worth noting that the percentage of free polyphenols was lower in CUBTAM (encapsulated) than in EXT (non-encapsulated) ones, and remained fairly constant along different in vitro digestion phases. These results were also expected because the initial amount of polyphenols in the encapsulated sample was lower owing to the encapsulated efficiency (over 50%). According to Ydjedd et al. [32], the properties of encapsulating material play a significant role in enhancing the entrapment efficiency and controlled release of the core compounds. They reported a slow release of some phenolics (gallic acid, p-coumaric acid, and kaempferol) from the microcapsules and a period of more than 3 h in the intestinal phase (neutral medium) has been necessary for complete release of these compounds, when the encapsulating material was completely degraded [32].

The present study is the first to report the proportion of cubosome encapsulated polyphenols released after each phase of in vitro digestion, demonstrating the potential of cubosomes to protect bioactive compounds in their matrix. Similarly, reduction of the degradation in cubosome encapsulated bioactive antimicrobial peptide has been reported, showing resistance towards the enzymatic degradation [18]. Cubosomes have a high viscosity which hinders the diffusion of polyphenols into the release medium and slows the entry of water, which sustains the slow release profile [47]. The rate of release controlled by the structure also depends both on the partition coefficient and on the diffusion of the drug through the hydrocarbon tail region [48].

### 3.4. Physicochemical Properties of Yoghurt Fortified with CUBTAM

The addition of 5%, 10% and 15% CUBTAM to yoghurt was associated with a small but statistically significant fall in pH and reduced syneresis (Table 2). This can be explained by the use of the freeze-drying treatment to prepare the powder for the cubosome, which would result in an increase in total dry solids, which in turn would increase the water holding capacity, reduce porosity and reduce the syneresis.

Viscosity of yoghurt increased with the increase of the concentration of CUBTAM, showing significant (*p* < 0.05) differences across the yoghurt samples (Table 2). Within the same % CUBTAM fortification, there was no significant (*p* > 0.05) difference in viscosity between PRE and POS. Based on the Oswald–de Waele power law model, yoghurts fortified with CUBTAM made from both fermentation processes can be considered as non-Newtonian fluids with shear-thinning behaviour due to the flow behaviour index (n) below 1. The breakage of bonds between the protein aggregates as a consequence of shear stress led to the pseudoplastic behaviour of the yoghurt samples [49]. The consistency index (K) and flow behaviour index (n) of yoghurts were not significantly influenced by the fermentation process, whereas the increase of encapsulated powder concentration led to the increase of K and decrease of *n* values. The increase of K value might be attributed to the water holding capacity, caused by the addition of powder.

The elastic modulus of all yoghurts was very low, indicating the same relatively weak structure with or without CUBTAM (Table 2).

### 3.5. Total Phenol Content, Antioxidant Activity and Release of Polyphenol Compounds in Yoghurt Fortified with CUBTAM during Digestion

**Figure 4:**
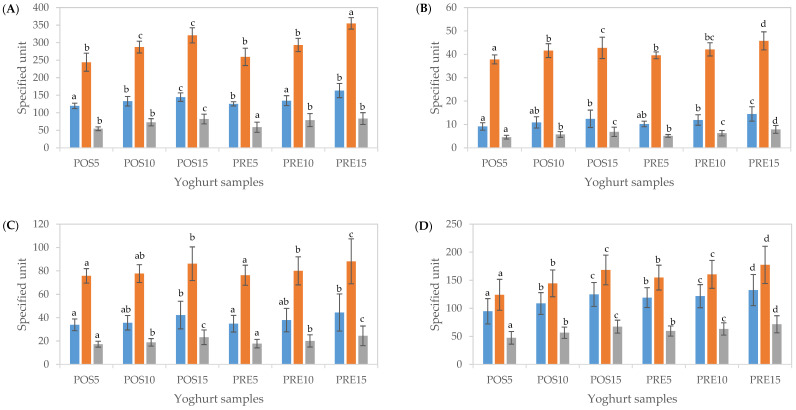
Total phenolic content (TPC) and antioxidant activity (CUPRAC and FRAP assay) of yoghurts fortified with cubosome containing tamarillo extract before digestion (**A**), after oral (**B**), gastric (**C**) and intestinal (**D**) phases of in vitro digestion. The units of TPC (■), CUPRAC (■) and FRAP (■) were mg GAE/100 g yoghurt, μmol TEAC/100 g yoghurt and μmol TEAC/100 g yoghurt, respectively. Data are presented as mean and error bar (standard deviation) (*n* = 3). Different alphabets indicate statistical difference (*p* < 0.05) for each assay.

In a yoghurt matrix, catalase and super oxidase enzymes, casein as well as lactic acid bacteria which have antioxidant properties are present [50]. Without digestion, as expected, the addition of CUBTAM led to a dose-dependent increase in TPC and total antioxidant capacity; i.e., as expected, a higher level of fortification led to a higher TPC as well as antioxidant activity (*p* < 0.05). Furthermore, fortification in PRE resulted in higher TPC and antioxidant activity than in POS at the same concentration (Figure 4A).

After the oral phase, TPC and total antioxidant capacity determined using CUPRAC and FRAP assays in both POS and PRE samples at all fortified concentrations reduced by 7.6–8.9%, 12.9–15.5% and 7.8–9.4%, respectively, compared to undigested samples (Figure 4B). After the gastric phase, significant increases in TPC and total antioxidant capacity were obtained compared to those of the oral phase (up to 3-, 2- and 3-fold, respectively), (*p* < 0.05) (Figure 4C). After simulated intestinal digestion, further significant increases in TPC (64–71%) were observed (*p* < 0.05). Furthermore, total antioxidant activity resulted in an additional 39–50% and 63–70% increase for CUPRAC and FRAP, respectively (Figure 4D). These results were in line with the measures of TPC and antioxidant activity of encapsulated tamarillo extracts (CUBTAM) (Figure 3) as well as polyphenol concentration determined by LC-MS (Table 1), in which the TPC and antioxidant activity increased greatly during the gastric and intestinal phases of in vitro digestion. Previous research showed that the antioxidant activity of the yoghurt samples containing encapsulated phenolics was increased due to the controlled release of the phenolic components from the encapsulation network [50]. Some researchers have considered the Folin–Ciocalteau assay as an antioxidant capacity test since this assay not only measures phenolic compounds but also the total reducing capacity of a sample and hence [42]. This may explain the difference in polyphenols content measured by LC-MS and by the colorimetric tests. Considering the pH conditions of the total antioxidant capacity assays performed in this study, the CUPRAC assay (pH 7.0) could be more appropriate for evaluating total antioxidant activity after the oral and intestinal phases, while the FRAP assay (pH 3.6) could be more suitable to assess total antioxidant activity after the gastric phase.

The data in Table 3 are described as a percentage of each digestion phase in the supernatant compared to the content of the undigested sample. The TPC and antioxidant activities measured by CUPRAC and FRAP between CUBTAM and yoghurt fortified with CUBTAM were relatively similar at each phase of the digestion simulation (Table 3), demonstrating that yoghurt was a suitable carrier of cubosome without significant interference. For the oral phase, the TPC released and antioxidant activities in CUBTAM fortified yoghurt were higher than that for the CUBTAM itself by 1.5–1.7, 1.2–1.5 and 1.1–1.4 times, respectively. For the gastric phase, the amount of TPC released for CUBTAM was slightly higher than the fortified yoghurts, whereas the opposite trend was observed for the antioxidant activities. For the intestinal phase, the TPC and FRAP of CUBTAM were lower than that for CUBTAM fortified yoghurts, whereas, for the CUPRAC, the CUBTAM showed a similar value to PRE5, which were both much higher compared to the rest of the samples. Overall, cubosomes containing tamarillo extract showed effective protection for polyphenols in the oral and gastric phases.

The differences in the LC-MS profiles between PRE and POS were significant (*p* < 0.05). Major polyphenols in tamarillo were also detected in CUBTAM fortified yoghurts (both from PRE and POS) before digestion (Table S2). Thus, the yoghurt matrix as well as encapsulation had helped to retain these individual polyphenols during processing. Encapsulation of bioactive compounds promoted lower loss of polyphenols under refrigerated conditions [51]. At the same concentration of CUBTAM yoghurt, addition of tamarillo polyphenol loaded-cubosomes prior to fermentation was associated with a higher concentration of major polyphenols than addition post fermentation. The concentrations of chlorogenic acid, kaempferol rutinoside and delphinidin rutinoside (accounted for over 65% of the total polyphenol content in yoghurts) were higher for pre-fermentation versus the post fermentation approach (Table S2). Delphinidin rutinoside was the dominant anthocyanin in fortified yoghurts that was in agreement with the main anthocyanin in Laird’s Large tamarillo pulp reported in our previous study [13]. The results showed that the fermentation process appeared to have little impact on anthocyanins present in both yoghurts, which might be due to the encapsulation of anthocyanins.

The yields of polyphenols were associated with the extractability of polyphenols from the original tamarillo extract. According to Sun-Waterhouse, Zhou and Wadhwa [52], during fermentation, the yoghurt starter cultures could transform the polyphenols into other forms/types of compounds, e.g., via flavonoid glycosides hydrolysis or C-ring cleavage. Such a conversion may result in the deactivation of bioactive compounds or activation of previously inactive compounds, e.g., polyphenol glycosides are hydrolyzed into their aglycones of higher free radical scavenging ability, and procyanidins break down to flavan-3-ols or to smaller molecular phenolic acids. Acidity of yoghurt may have induced acid hydrolysis of polyphenols, hence this could explain an increased amount of hydroxycinnamates such as caffeic acid, chlorogenic acid, ferulic acid and p-coumaric acid in the fortified yoghurts. Sun-Waterhouse, Zhou and Wadhwa [53] stated that the yields of hydroxycinnamic acids and flavonols detected might be dependent on the extractability of these polyphenols from different product matrices.

Despite the importance of the recovery in each digestion phase, bioactive compounds will need to be released from their food matrix and reach the intestine where they can be absorbed and be metabolised [54]. The release rate during in vitro digestion has been considered as an indicator to assess the effectiveness of compound carriers [55]. In general, non-encapsulated phenolic compounds in drinking yoghurt were highly degraded after digestion [56] while microencapsulated formulation showed the ability to preserve the antioxidant activity of extract in yoghurt when compared with the free form [51,57]. The amount of individual polyphenol in yoghurt samples was significantly different (*p* < 0.05) after each phase of in vitro digestion. The quantity of polyphenols released at each stage is dependent on the time at each phase, pH and the concentration of CUBTAM (Table S3). Most polyphenol components were detected in both PRE and POS in each digestion phase. The oral digestion lasts for a few minutes; the encapsulated polyphenol in yoghurts release from oral digestion was significantly lower than the gastric (post to 2 h) and intestinal (post to 3 h) simulated digests. These data are in line with the findings from Section 3.3 that encapsulating polyphenol in cubosome particles could effectively protect the bioactive compounds from the gastric enzymes and facilitate the utilization of polyphenols in the human body. Studies on the metabolism of bioactive compounds in the humans have shown that bioactives are mainly metabolised by a large number of small and large intestinal bacteria, and the metabolites are absorbed into the human blood [58]. Therefore, for the polyphenols to be absorbed by the human body or to be active in the microbiome of the small and large intestines, the polyphenols should be protected in encapsulated form until completely released in the intestinal tract. When the polyphenol capsules were present in the simulated oral phase, the concentration of all bioactive compounds was low, indicating good retention in the cubosomes. Furthermore, compared to undigested samples, a significantly (*p* < 0.05) lower amount of most polyphenols (percentage loss < 10%) were released during the oral phase (except for epicatechin, rutin and kaempferol rutinoside). The findings provide evidence that cubosomes protect bioactive compounds from interaction with, for example, milk proteins and digestive enzymes, reducing the risk of polyphenol degradation.

To our knowledge, most of reports of the loads of cubosomes are for proteins or small molecules such as drugs while research about encapsulation of hydrophilic polyphenols, mainly presented in tamarillo, using the cubosome is limited. Effects of the digestion process on properties and stability of cubosome as well as application of cubosome in food are still scarce. Hence, a strength of this study is it is the first attempt to encapsulate polyphenols from tamarillo and has demonstrated the proof of principle that this technique can be used to fortify yoghurt with a fruit extract. Entrapment efficiency was greater than 50% and, together with enhancement of antioxidant effects, stability and bioavailability of polyphenols in vivo and in vitro [2], the results showed the potential of cubosome to minimize degradation of polyphenols and contribute to controlled release of these and other bioactives during digestion. Application of encapsulated polyphenols into yoghurt did not significantly change texture and rheology of yoghurts when compared to the control, except for 15% fortification, where higher texture and rheology values were observed. However, there are still challenges about applications of cubosomes, including a deeper understanding of the stabilizer and possible cytotoxicity. In the future interaction between yoghurt components (mainly protein), starter culture and encapsulated bioactives should be evaluated with longer intestinal digestion and possible effects on the microbiota explored. According to Wei et al. [21], monoolein has been easily hydrolysed to free oleic acid due to the presence of pancreatic lipase and bile salt; therefore, it can be assumed that cubosome particles (with 60% of monoolein in components) can only be degraded in intestinal phase. However, degradation or stability of cubosome after each phase of digestion should be evaluated to ensure the safety, effectiveness and acceptability to the consumer of this approach. It is a limitation that we did not validate the correct encapsulation of the tamarillo polyphenols in the cubosome. In future work, we could confirm the encapsulation with confocal Raman/FTIR microscopy.

## 4. Conclusions

This study demonstrated the proof-of-principle that tamarillo polyphenols could be effectively encapsulated by cubosome nanoparticles with relatively high loading efficiency and preservation of high antioxidant activity. Compared to the unencapsulated extract, cubosomal encapsulation provided a protective effect to the tamarillo polyphenols under simulated gastrointestinal conditions, exhibiting good free polyphenol concentrations at the end of the intestinal phase. A cubosomal system was employed for the delivery of tamarillo polyphenols via yoghurt, and the addition of encapsulated bioactive improved the physicochemical and nutritional properties of yoghurt. The addition of CUBTAM at increasing concentrations successfully increased the concentration of polyphenols, TPC and antioxidant activity of yoghurts, with controlled stability during digestion, suggesting that polyphenols with enhanced bioavailability could be delivered in a dose-controlled manner. This research informs application of cubosome encapsulation to fortification of food products, for example both water-soluble and lipid-soluble vitamins and carotenoids (β-carotene). However, although the components of cubosomes (monoolein and Pluronic F127) are listed as “generally recognised as safe” (GRAS) by the FDA and approved in principle, further investigations should be carried out before sensory testing or consumption by humans as a food.

## Figures and Tables

**Table 1:** Phenolics and anthocyanins in tamarillo extract and tamarillo polyphenol loaded-cubosome released during three stages of in vitro digestion.

Bioactive/Phases	Tamarillo Extract			Tamarillo Polyphenol Loaded-Cubosome		
	Oral	Gastric	Intestinal	Oral	Gastric	Intestinal
*Phenolics*						
Gallic Acid	3.86 ± 0.84 ^a^	46.5 ± 6.02 ^b^	20.4 ± 1.86 ^c^	4.51 ± 0.79 ^a^	8.57 ± 0.85 ^a^	52.9 ± 11.7 ^d^
Catechin	24.5 ± 5.01 ^a^	27.2 ± 7.93 ^a^	25.4 ± 6.09 ^a^	6.37 ± 0.52 ^b^	23.5 ± 4.09 ^a^	16.3 ± 0.39
Caffeic Acid	24.1 ± 4.55 ^a^	31.4 ± 3.90 ^b^	21.9 ± 7.06 ^a^	81.2 ± 15.2 ^c^	2.73 ± 0.91 ^d^	8.03 ± 0.35 ^e^
Chlorogenic Acid	9.40 ± 1.24 ^a^	67.7 ± 12.3 ^b^	4.82 ± 1.12 ^c^	4.99 ± 0.44 ^c^	9.33 ± 0.43 ^a^	28.8 ± 4.02 ^d^
Epicatechin	24.2 ± 6.22 ^a^	55.2 ± 10.3 ^b^	13.9 ± 1.10 ^c^	9.42 ± 0.49 ^d^	16.6 ± 2.62 ^c^	16.3 ± 3.66 ^ac^
p-coumaric acid	38.8 ± 8.36 ^a^	39.4 ± 6.29 ^a^	4.14 ± 0.36 ^b^	31.1 ± 1.16 ^c^	16.2 ± 0.97 ^d^	23.2 ± 4.20 ^e^
Ferulic Acid	5.55 ± 0.98 ^a^	13.4 ± 2.62 ^b^	17.8 ± 2.64 ^b^	54.4 ± 8.75 ^c^	3.42 ± 0.98 ^a^	32.3 ± 7.54 ^d^
Rutin	31.0 ± 6.09 ^a^	38.7 ± 7.40 ^a^	23.0 ± 5.96 ^b^	16.5 ± 1.39 ^bc^	14.7 ± 2.69 ^c^	24.0 ± 5.79 ^b^
Kaempferol rutinoside	32.1 ± 8.67 ^a^	37.6 ± 5.87 ^a^	15.5 ± 2.00 ^b^	21.0 ± 3.76 ^c^	21.5 ± 4.49 ^c^	29.1 ± 4.72 ^ac^
Isorhamnetin rutinoside	8.21 ± 1.04 ^a^	10.3 ± 1.08 ^b^	9.81 ± 1.08 ^ab^	3.76 ± 0.52 ^c^	10.2 ± 2.20 ^b^	9.43 ± 1.89 ^ab^
Kaempferol	43.8 ± 12.1 ^a^	20.4 ± 5.01 ^b^	5.83 ± 0.70 ^c^	29.6 ± 4.51 ^b^	20.2 ± 5.21 ^b^	33.1 ± 3.25 ^d^
*Anthocyanins*						
Delphinidin rutinoside	10.1 ± 0.76 ^a^	24.6 ± 5.97 ^b^	7.87 ± 0.40 ^c^	5.30 ± 0.32 ^d^	10.3 ± 1.16 ^a^	19.4 ± 1.14 ^b^
Cyanidin rutinoside	14.3 ± 1.26 ^a^	35.9 ± 6.17 ^b^	4.31 ± 0.73 ^c^	8.68 ± 0.55 ^d^	17.1 ± 6.90 ^a^	25.2 ± 0.09 ^e^
Pelargonidin rutinoside	20.6 ± 1.49 ^a^	48.7 ± 5.26 ^b^	6.16 ± 0.31 ^c^	10.3 ± 1.46 ^d^	14.6 ± 2.80 ^d^	27.8 ± 1.36 ^e^

Results are expressed as % with respect to the initial concentration. Data are expressed as Mean ± SD (*n* = 3). Different letters of the alphabet superscripts indicate statistical difference (*p* < 0.05) across each row.

**Table 2:** Physicochemical properties of yoghurt fortified with CUBTAM (5, 10 and 15%) in PRE and POS. Control yoghurt contained no CUBTAM.

Parameters/Samples	Control	POS5	POS10	POS15	PRE5	PRE10	PRE15
pH	4.35 ± 0.03 ^a^	4.27 ± 0.02 ^b^	4.14 ± 0.01 ^c^	4.09 ± 0.02 ^d^	4.30 ± 0.00 ^a^	4.18 ± 0.01 ^c^	4.10 ± 0.02 ^cd^
Syneresis (%)	29.3 ± 0.91 ^a^	28.8 ± 1.48 ^a^	27.1 ± 1.53 ^ab^	26.6 ± 1.91 ^b^	28.7 ± 1.36 ^a^	27.9 ± 1.70 ^b^	26.9 ± 1.52 ^b^
*Textural parameters*							
Firmness (N)	1.027 ± 0.005 ^a^	1.031 ± 0.004 ^a^	1.034 ± 0.002 ^ab^	1.042 ± 0.008 ^b^	1.033 ± 0.004 ^a^	1.035 ± 0.003 ^ab^	1.045 ± 0.006 ^b^
Consistency (N.sec)	16.08 ± 0.004 ^a^	16.12 ± 0.029 ^a^	16.21 ± 0.021 ^b^	17.19 ± 0.068 ^c^	16.09 ± 0.102 ^a^	16.23 ± 0.039 ^b^	17.22 ± 0.065 ^d^
Cohesiveness (N)	−0.005 ± 0.003 ^a^	−0.007 ± 0.002 ^a^	−0.011 ± 0.004 ^ab^	−0.014 ± 0.005 ^b^	−0.008 ± 0.003 ^a^	−0.010 ± 0.001 ^ab^	−0.016 ± 0.007 ^b^
*Rheological parameters*							
Consistency coefficient (K, Pa.s)	<0.005 ^a^	0.009 ± 0.003 ^b^	0.010 ± 0.003 ^b^	0.014 ± 0.007 ^b^	0.008 ± 0.02 ^b^	0.012 ± 0.005 ^b^	0.015 ± 0.008 ^b^
Flow behaviours index (n)	0.781 ± 0.012 ^a^	0.729 ± 0.022 ^a^	0.658 ± 0.010 ^b^	0.604 ± 0.018 ^c^	0.735 ± 0.014 ^a^	0.678 ± 0.020 ^b^	0.642 ± 0.015 ^b^
Viscosity at 350 s^−1^ (Pa.s)	0.026 ± 0.000 ^a^	0.030 ± 0.000 ^b^	0.033 ± 0.001 ^c^	0.041 ± 0.001 ^d^	0.032 ± 0.000 ^b^	0.037 ± 0.002 ^cd^	0.045 ± 0.003 ^d^
Elastic modulus (Pa)	N/A	0.003 ± 0.000 ^a^	0.005 ± 0.000 ^b^	0.005 ± 0.001 ^b^	0.003 ± 0.000 ^a^	0.004 ± 0.001 ^b^	0.006 ± 0.002 ^b^

N/A: not applicable. Data are expressed as Mean ± SD (*n* = 3). Different alphabetic superscripts indicate statistical difference (*p* < 0.05) across each row. CUBTAM: tamarillo polyphenols loaded-cubosomes. POS5, POS10, POS15: addition of 5%, 10%, 15% of CUBTAM post to fermentation process, respectively. PRE5, PRE10 and PRE15: addition of 5%, 10%, 15% of CUBTAM prior to fermentation process, respectively.

**Table 3:** Measures of total phenolic content and antioxidant activity of supernatant of cubosome encapsulated tamarillo and yoghurt fortified with encapsulated tamarillo during in vitro digestion.

Phases	Samples	TPC	CUPRAC	FRAP
Oral	CUBTAM	5.07	10.55	6.97
	POS5	7.64	15.50	8.38
	POS10	8.19	14.47	7.83
	POS15	8.59	13.34	8.33
	PRE5	8.14	15.25	8.67
	PRE10	8.86	14.36	7.93
	PRE15	8.87	12.89	9.45
Gastric	CUBTAM	29.13	22.77	24.70
	POS5	28.34	31.04	31.84
	POS10	26.79	27.05	25.88
	POS15	29.20	26.86	28.24
	PRE5	27.75	29.41	30.18
	PRE10	28.18	27.29	25.39
	PRE15	27.19	24.84	29.25
Intestinal	CUBTAM	77.03	58.53	65.27
	POS5	79.20	50.83	87.22
	POS10	81.72	50.27	77.47
	POS15	86.30	52.49	81.92
	PRE5	94.90	59.64	101.19
	PRE10	90.41	54.74	79.94
	PRE15	81.08	49.99	85.62

Data are presented as % of the sample before digestion. CUBTAM: tamarillo polyphenols loaded-cubosomes. POS5, POS10, POS15: addition of 5%, 10%, 15% of CUBTAM post to fermentation process, respectively. PRE5, PRE10 and PRE15: addition of 5%, 10%, 15% of CUBTAM prior to fermentation process, respectively.

## Data Availability

All data are contained within the article and supplementary material.

## Supplementary Materials

The following supporting information can be downloaded at: https://www.mdpi.com/article/10.3390/antiox11030520/s1, Table S1: Particle size and polydispersity index of CUB and CUBTAM, Table S2: Concentrations (μg/g FW) of individual polyphenols in yoghurts fortified with CUBTAM before in vitro digestion, Table S3: Concentrations (μg/g FW) of individual polyphenols in yoghurts fortified with CUBTAM after each step of in vitro digestion, Figure S1: Size distribution of CUB (♦) and CUBTAM (⬤), Figure S2: Concentration of polyphenols added into cubosome ((■) and concentration of polyphenols present in the supernatant (■).
