# Supplementary material for: Tamarillo Polyphenols Encapsulated-Cubosome: Formation, Characterization, Stability during Digestion and Application in Yoghurt
Source: Antioxidants (Basel). 2022 Mar 8;11(3):520. doi: 10.3390/antiox11030520 (PMC8944466; doi:10.3390/antiox11030520)
Supplement: Supplementary file 1 [file antioxidants-11-00520-s001.zip › antioxidants-1609098-supplementary.pdf]

Table S1. Particle size and polydispersity index of CUB and CUBTAM

Parameters	CUB	CUBTAM
Z-average (nm)	270.9 ± 5.61	322.4 ± 7.27
PDI	0.237	0.272

* Data are presented as Mean ± SD (n ≥ 3). CUB: cubosome particle, CUBTAM: tamarillo polyphenols loaded-cubosome. PDI polydispersity index

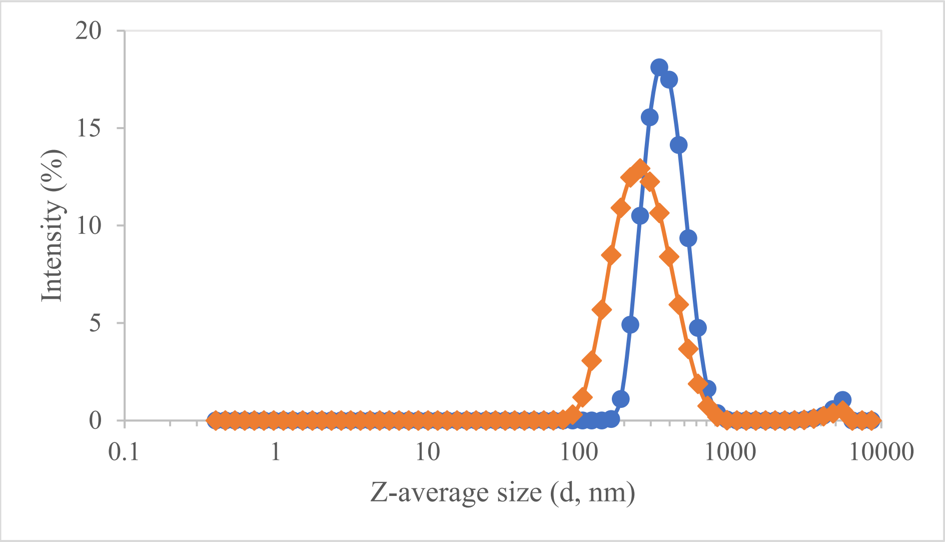

Figure S1. Size distribution of CUB (◆) and CUBTAM (●)

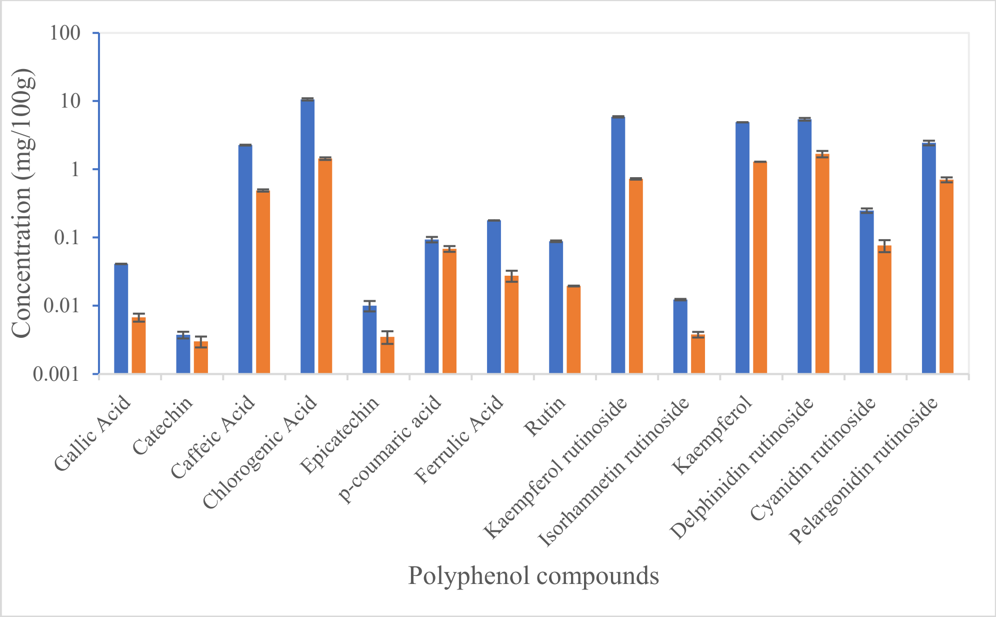

Figure S2. Concentration of polyphenols added into cubosome (■) and concentration of polyphenols present in the supernatant (■)

Table S2. Concentrations (μg/g yoghurt) of individual polyphenols in yoghurts fortified with CUBTAM before *in vitro* digestion.

Polyphenols/phases	Undigested					
	POS5	POS10	POS15	PRE5	PRE10	PRE15
Phenolics						
Gallic Acid	0.011 ± 0.001^{a}	0.028 ± 0.002^{b}	0.068 ± 0.004^{c}	0.012 ± 0.001^{a}	0.050 ± 0.002^{d}	0.070 ± 0.001^{c}
Catechin	0.002 ± 0.001^{a}	0.004 ± 0.002^{b}	0.005 ± 0.002^{b}	0.004 ± 0.002^{b}	0.004 ± 0.003^{b}	0.004 ± 0.003^{b}
Caffeic acid	0.174 ± 0.032^{a}	0.336 ± 0.091^{b}	0.761 ± 0.168^{c}	0.163 ± 0.008^{a}	0.482 ± 0.078^{b}	0.700 ± 0.027^{c}
Chlorogenic acid	0.848 ± 0.017^{a}	1.696 ± 0.055^{b}	3.392 ± 0.138^{c}	1.275 ± 0.056^{b}	2.646 ± 0.055^{d}	3.821 ± 0.097^{c}
Epicatechin	0.004 ± 0.002^{a}	0.011 ± 0.007^{b}	0.014 ± 0.012^{c}	0.002 ± 0.000^{a}	0.003 ± 0.003^{a}	0.003 ± 0.001^{a}
p-coumaric acid	0.030 ± 0.010^{a}	0.064 ± 0.020^{b}	0.107 ± 0.025^{c}	0.050 ± 0.014^{b}	0.061 ± 0.030^{b}	0.086 ± 0.006^{d}
Ferulic acid	0.037 ± 0.003^{a}	0.106 ± 0.012^{b}	0.132 ± 0.015^{c}	0.053 ± 0.008^{d}	0.162 ± 0.014^{e}	0.159 ± 0.037^{e}
Rutin	0.047 ± 0.014^{a}	0.051 ± 0.023^{a}	0.060 ± 0.031^{a}	0.019 ± 0.007^{b}	0.029 ± 0.015^{b}	0.039 ± 0.012^{c}
Kaempferol rutinoside	0.333 ± 0.062^{a}	0.547 ± 0.009^{b}	0.847 ± 0.023^{c}	0.345 ± 0.018^{a}	0.644 ± 0.017^{b}	0.860 ± 0.032^{c}
Isorhamnetin rutinoside	0.103 ± 0.049^{a}	0.136 ± 0.003^{b}	0.136 ± 0.003^{b}	0.134 ± 0.000^{ab}	0.137 ± 0.004^{b}	0.135 ± 0.001^{c}
Kaempferol	0.058 ± 0.016^{a}	0.236 ± 0.031^{b}	0.363 ± 0.026^{c}	0.216 ± 0.015^{b}	0.348 ± 0.058^{c}	0.597 ± 0.016^{d}
Anthocyanins						
Delphinidin rutinoside	0.171 ± 0.008^{a}	0.249 ± 0.004^{b}	0.419 ± 0.010^{c}	0.156 ± 0.005^{a}	0.378 ± 0.019^{bc}	0.443 ± 0.057^{c}
Cyanidin rutinoside	0.023 ± 0.014^{a}	0.041 ± 0.010^{b}	0.061 ± 0.031^{c}	0.025 ± 0.011^{a}	0.073 ± 0.040^{c}	0.094 ± 0.040^{d}
Pelargonidin rutinoside	0.067 ± 0.019^{a}	0.100 ± 0.035^{b}	0.228 ± 0.120^{c}	0.136 ± 0.060^{b}	0.164 ± 0.082^{bc}	0.283 ± 0.161^{c}

* Data are expressed as Mean ± SD (n = 3). Different alphabets superscripts indicate statistical difference ($p < 0.05$) across each row. No polyphenols were detected in the control yoghurt. POS5, POS10, POS15: addition of 5, 10, 15% of CUBTAM post to fermentation process, respectively. PRE5, PRE10 and PRE15: addition of 5, 10, 15% of CUBTAM prior to fermentation process, respectively.

Table S3. Concentrations (μg/g yoghurt) of individual polyphenols in yoghurts fortified with CUBTAM after each step of *in vitro* digestion

Polyphenols/phases	Oral					
	POS5	POS10	POS15	PRE5	PRE10	PRE15
Phenolics						
Gallic Acid	0.001 ± 0.000^{a}	0.001 ± 0.000^{a}	0.001 ± 0.001^{a}	0.001 ± 0.000^{a}	$< 0.0005^{a}$	$< 0.0005^{a}$
Catechin	0.001 ± 0.000^{a}	0.001 ± 0.001^{a}	0.001 ± 0.000^{a}	$< 0.0005^{a}$	0.001 ± 0.002^{a}	0.001 ± 0.001^{a}
Caffeic acid	0.008 ± 0.004^{a}	0.012 ± 0.003^{b}	0.052 ± 0.011^{c}	0.009 ± 0.005^{a}	0.018 ± 0.004^{b}	0.020 ± 0.004^{b}
Chlorogenic acid	0.006 ± 0.002^{a}	0.005 ± 0.002^{a}	0.014 ± 0.010^{b}	0.005 ± 0.001^{a}	0.006 ± 0.004^{a}	0.010 ± 0.001^{c}
Epicatechin	0.001 ± 0.000^{a}	0.001 ± 0.000^{a}	0.001 ± 0.001^{a}	0.001 ± 0.000^{a}	0.001 ± 0.001^{a}	0.001 ± 0.000^{a}
p-coumaric acid	0.012 ± 0.005^{a}	0.010 ± 0.002^{a}	0.009 ± 0.006^{a}	0.015 ± 0.006^{b}	0.024 ± 0.011^{bc}	0.037 ± 0.013^{c}
Ferulic acid	0.004 ± 0.003^{a}	0.005 ± 0.001^{a}	0.006 ± 0.002^{a}	0.004 ± 0.001^{a}	0.005 ± 0.002^{a}	0.004 ± 0.002^{a}
Rutin	0.007 ± 0.003^{a}	0.012 ± 0.005^{ab}	0.014 ± 0.005^{b}	0.007 ± 0.003^{a}	0.009 ± 0.005^{a}	0.013 ± 0.007^{ab}
Kaempferol rutinoside	0.101 ± 0.021^{a}	0.217 ± 0.008^{b}	0.349 ± 0.036^{c}	0.124 ± 0.018^{a}	0.250 ± 0.012^{b}	0.354 ± 0.043^{c}
Isorhamnetin rutinoside	0.030 ± 0.001^{a}	0.030 ± 0.000^{a}	0.030 ± 0.001^{a}	0.029 ± 0.000^{a}	0.030 ± 0.001^{a}	0.030 ± 0.000^{a}
Kaempferol	0.001 ± 0.001^{a}	0.002 ± 0.001^{ab}	0.003 ± 0.001^{b}	0.002 ± 0.001^{ab}	0.004 ± 0.003^{b}	0.003 ± 0.001^{b}
Anthocyanins						
Delphinidin rutinoside	n.d	n.d	n.d	n.d	n.d	n.d
Cyanidin rutinoside	n.d	n.d	n.d	n.d	n.d	n.d
Pelargonidin rutinoside	n.d	n.d	n.d	n.d	n.d	n.d

* n.d: not detected. Data are expressed as Mean ± SD (n = 3). Different alphabets superscripts indicate statistical difference ($p < 0.05$) across each row. No polyphenols were detected in the control yoghurt. POS5, POS10, POS15: addition of 5, 10, 15% of CUBTAM post to fermentation process, respectively. PRE5, PRE10 and PRE15: addition of 5, 10, 15% of CUBTAM prior to fermentation process, respectively.

Table S3. Concentrations (µg/g yoghurt) of individual polyphenols in yoghurts fortified with CUBTAM after each step of *in vitro* digestion (Cont.)

Polyphenols/phases	Gastric					
	POS5	POS10	POS15	PRE5	PRE10	PRE15
Phenolics						
Gallic Acid	0.006 ± 0.001^{a}	0.011 ± 0.001^{b}	0.021 ± 0.001^{c}	0.002 ± 0.001^{d}	0.018 ± 0.001^{c}	0.023 ± 0.002^{c}
Catechin	0.002 ± 0.002^{a}	0.002 ± 0.000^{a}	0.001 ± 0.001^{a}	0.001 ± 0.000^{a}	0.002 ± 0.001^{a}	0.002 ± 0.001^{a}
Caffeic acid	0.040 ± 0.002^{a}	0.119 ± 0.003^{b}	0.191 ± 0.010^{c}	0.015 ± 0.001^{d}	0.112 ± 0.004^{b}	0.147 ± 0.007^{e}
Chlorogenic acid	0.234 ± 0.088^{a}	0.256 ± 0.116^{a}	0.425 ± 0.171^{b}	0.113 ± 0.004^{c}	0.129 ± 0.028^{c}	0.161 ± 0.048^{ac}
Epicatechin	0.001 ± 0.001^{a}	0.004 ± 0.001^{b}	0.008 ± 0.003^{c}	0.002 ± 0.001^{a}	0.001 ± 0.000^{a}	0.002 ± 0.001^{ab}
p-coumaric acid	0.022 ± 0.010^{a}	0.051 ± 0.020^{b}	0.049 ± 0.021^{b}	0.009 ± 0.005^{c}	0.009 ± 0.006^{c}	0.012 ± 0.006^{c}
Ferulic acid	0.018 ± 0.004^{a}	0.038 ± 0.013^{b}	0.043 ± 0.022^{bc}	0.034 ± 0.015^{b}	0.059 ± 0.013^{c}	0.106 ± 0.014^{d}
Rutin	0.013 ± 0.005^{a}	0.014 ± 0.004^{a}	0.019 ± 0.009^{b}	0.013 ± 0.007^{a}	0.011 ± 0.006^{a}	0.015 ± 0.006^{a}
Kaempferol rutinoside	0.243 ± 0.012^{a}	0.454 ± 0.174^{b}	0.529 ± 0.204^{bc}	0.292 ± 0.039^{a}	0.517 ± 0.096^{b}	0.637 ± 0.026^{c}
Isorhamnetin rutinoside	0.060 ± 0.000^{a}	0.065 ± 0.000^{a}	0.065 ± 0.001^{a}	0.061 ± 0.001^{a}	0.065 ± 0.000^{a}	0.064 ± 0.001^{a}
Kaempferol	0.018 ± 0.002^{a}	0.032 ± 0.020^{b}	0.100^{c} ± 0.010^{c}	0.032 ± 0.006^{b}	0.044 ± 0.020^{d}	0.035 ± 0.014^{bd}
Anthocyanins						
Delphinidin rutinoside	0.085 ± 0.018^{a}	0.114 ± 0.018^{b}	0.251 ± 0.003^{c}	0.068 ± 0.038^{a}	0.152 ± 0.066^{b}	0.251 ± 0.008^{c}
Cyanidin rutinoside	n.d	0.001 ± 0.000^{a}	0.009 ± 0.003^{b}	n.d	0.022 ± 0.013^{c}	0.060 ± 0.035^{d}
Pelargonidin rutinoside	0.014 ± 0.002^{a}	0.022 ± 0.001^{b}	0.070 ± 0.007^{c}	0.041 ± 0.005^{d}	0.097 ± 0.001^{e}	0.110 ± 0.006^{f}

* n.d: not detected. Data areexpressed as Mean ± SD (n = 3). Different alphabets superscripts indicate statistical difference ($p < 0.05$) across each row. No polyphenols were detected in the control yoghurt. POS5, POS10, POS15: addition of 5, 10, 15% of CUBTAM post to fermentation process, respectively. PRE5, PRE10 and PRE15: addition of 5, 10, 15% of CUBTAM prior to fermentation process, respectively.

Table S3. Concentrations (μg/g yoghurt) of individual polyphenols in yoghurts fortified with CUBTAM after each step of *in vitro* digestion (Cont.)

Polyphenols/phases	Intestinal					
	POS5	POS10	POS15	PRE5	PRE10	PRE15
Phenolics						
Gallic Acid	0.004 ± 0.002^{a}	0.016 ± 0.001^{b}	0.025 ± 0.001^{c}	0.002 ± 0.001^{a}	0.014 ± 0.004^{b}	0.021 ± 0.005^{c}
Catechin	0.004 ± 0.003^{a}	0.003 ± 0.001^{ab}	0.004 ± 0.003^{a}	0.002 ± 0.001^{b}	0.002 ± 0.000^{b}	0.002 ± 0.001^{b}
Caffeic acid	0.042 ± 0.003^{a}	0.266 ± 0.066^{b}	0.494 ± 0.052^{c}	0.055 ± 0.011^{a}	0.268 ± 0.088^{b}	0.458 ± 0.043^{c}
Chlorogenic acid	0.342 ± 0.078^{a}	0.531 ± 0.111^{b}	0.694 ± 0.102^{c}	0.280 ± 0.029^{a}	0.560 ± 0.032^{b}	0.667 ± 0.189^{c}
Epicatechin	0.003 ± 0.001^{a}	0.002 ± 0.002^{a}	0.004 ± 0.002^{b}	0.003 ± 0.001^{a}	0.003 ± 0.002^{a}	0.003 ± 0.003^{b}
p-coumaric acid	0.042 ± 0.016^{a}	0.044 ± 0.010^{a}	0.063 ± 0.009^{b}	0.022 ± 0.011^{c}	0.034 ± 0.013^{bc}	0.044 ± 0.012^{a}
Ferulic acid	0.025 ± 0.011^{a}	0.031 ± 0.014^{ab}	0.043 ± 0.014^{b}	0.014 ± 0.006^{c}	0.053 ± 0.017^{b}	0.095 ± 0.018^{d}
Rutin	0.019 ± 0.008^{a}	0.014 ± 0.007^{a}	0.026 ± 0.010^{b}	0.018 ± 0.008^{a}	0.014 ± 0.005^{a}	0.042 ± 0.011c
Kaempferol rutinoside	0.174 ± 0.016^{a}	0.463 ± 0.021^{b}	0.725 ± 0.033^{cd}	0.267 ± 0.020^{a}	0.600 ± 0.128^{c}	0.755 ± 0.136^{d}
Isorhamnetin rutinoside	0.031 ± 0.001^{a}	0.037 ± 0.000^{b}	0.038 ± 0.001^{b}	0.032 ± 0.000^{a}	0.037 ± 0.000^{b}	0.037 ± 0.001^{b}
Kaempferol	0.013 ± 0.003^{a}	0.042 ± 0.007^{b}	0.065 ± 0.013^{c}	0.032 ± 0.003^{d}	0.061 ± 0.010^{c}	0.098 ± 0.012^{e}
Anthocyanins						
Delphinidin rutinoside	0.030 ± 0.015^{a}	0.178 ± 0.038^{b}	0.249 ± 0.009^{c}	0.032 ± 0.006^{a}	0.099 ± 0.036^{d}	0.314 ± 0.099^{e}
Cyanidin rutinoside	0.003 ± 0.001^{a}	0.006 ± 0.002^{b}	0.013 ± 0.001^{c}	$< 0.0005^{d}$	0.018 ± 0.001^{e}	0.016 ± 0.002^{e}
Pelargonidin rutinoside	0.054 ± 0.031^{a}	0.088 ± 0.029^{b}	0.134 ± 0.054^{c}	0.057 ± 0.032^{a}	0.065 ± 0.039^{ab}	0.148 ± 0.045^{c}

* Data was expressed as Mean ± SD (n = 3). Different alphabets superscripts indicate statistical difference ($p < 0.05$) across each row. No polyphenols were detected in the control yoghurt. POS5, POS10, POS15: addition of 5, 10, 15% of CUBTAM post (POS) fermentation process, respectively. PRE5, PRE10 and PRE15: addition of 5, 10, 15% of CUBTAM prior (PRE)o fermentation process, respectively.
